# State-of-the-art augmented NLP transformer models for direct and single-step retrosynthesis

**DOI:** 10.1038/s41467-020-19266-y

**Published:** 2020-11-04

**Authors:** Igor V. Tetko, Pavel Karpov, Ruud Van Deursen, Guillaume Godin

**Affiliations:** 1 Institute of Structural Biology, Helmholtz Zentrum München—Research Center for Environmental Health (GmbH), Ingolstädter Landstraße 1, D-85764 Neuherberg, Germany; 2 BIGCHEM GmbH, Valerystr. 49, D-85716 Unterschleißheim, Germany; 3 Firmenich International SA, D-Lab by Firmenich, Rue de la Bergère 7, CH-1242 Meyrin-Satigny, Switzerland

**Keywords:** Cheminformatics, Reaction mechanisms, Computational chemistry

## Abstract

We investigated the effect of different training scenarios on predicting the (retro)synthesis of chemical compounds using text-like representation of chemical reactions (SMILES) and Natural Language Processing (NLP) neural network Transformer architecture. We showed that data augmentation, which is a powerful method used in image processing, eliminated the effect of data memorization by neural networks and improved their performance for prediction of new sequences. This effect was observed when augmentation was used simultaneously for input and the target data simultaneously. The top-5 accuracy was 84.8% for the prediction of the largest fragment (thus identifying principal transformation for classical retro-synthesis) for the USPTO-50k test dataset, and was achieved by a combination of SMILES augmentation and a beam search algorithm. The same approach provided significantly better results for the prediction of direct reactions from the single-step USPTO-MIT test set. Our model achieved 90.6% top-1 and 96.1% top-5 accuracy for its challenging mixed set and 97% top-5 accuracy for the USPTO-MIT separated set. It also significantly improved results for USPTO-full set single-step retrosynthesis for both top-1 and top-10 accuracies. The appearance frequency of the most abundantly generated SMILES was well correlated with the prediction outcome and can be used as a measure of the quality of reaction prediction.

## Abstract (web summary)

Development of algorithms to predict reactant and reagents given a target molecule is key to accelerate retrosynthesis approaches. Here the authors demonstrate that applying augmentation techniques to the SMILE representation of target data significantly improves the quality of the reaction predictions.

## Introduction

To synthesize an organic compound is to solve a puzzle with many pieces and potentially several pieces missing. Here, the pieces are single reactions, and finding their sequential combination to create a final product is the retrosynthesis task.

The success of the logic of organic synthesis developed by Corey et al.^1,2^ triggered the development of computer programs aiming to find appropriate ways to synthesize a molecule. The first retrosynthesis program LHASA^2^ utilizes a template-based^3,4^ approach. Every template (rule, synthon) in a curated database of known transformations is sequentially applied to a target molecule, and then sets of reagents are selected according to a specified strategy. Reagents, in turn, undergo the same decompositions until a set of commercially available compounds is found. Retrosynthesis always has multiple routes—a retrosynthetic tree—ending with different starting materials. Thus, a practical algorithm for retrosynthesis has to solve not only the rule acquisition and selection problem but also has capabilities to effectively navigate this tree^5^, taking into account different strategies. These tasks relate directly to artificial intelligence strategies^6–8^.

Due to the difficulty of maintaining template databases, most projects dependent on them, including LHASA, did not become widely used tools. The only major exception is, perhaps, the program Synthia™ (previously CHEMATICA^9^) which is a successful commercial product. In the Synthia™ program, rules are automatically extracted from atom-mapped reaction examples^10^. However, there is an ambiguity in the mapping definition and, more importantly, the automatic rule does not take into account other undefined possible reactive centers in a molecule. Applying such transformations may result in molecules that fail to react as predicted, e.g., “out-of-scopes” and special care to filter out these cases has to be taken^5^. An alternative approach for the extraction of these rules is to apply a data-driven deep learning technique that corresponds to a machine learning approach where an algorithm (usually in the form of a neural network) is trained on the raw data. After the training finishes, the network contains all the implicitly encoded features (rules) of the corresponding input via its parameters. Works on reaction prediction outcomes^11^ and retrosynthesis^12,13^ showed the feasibility of a symbolic approach, where reactions are written as SMILES^14^ strings as in a machine translation. The product is written in the “source language”, whereas the set of reactants is written in the “target language”. For the “reaction translation” task both languages, however, are SMILES strings, having the same alphabet and grammar. The first works on symbolic (retro)synthesis^12,15^ were carried out with Seq2Seq^16^ models following robust and more easy to train natural language processing (NLP) transformer approaches^17,18^ that bring state-of-the-art results^11,19^. Meanwhile other approaches based on similarity^20^, convolutional^21–23^, and graphs^24,25^ show promising results.

The SMILES representation of molecules is ambiguous. Though the canonicalization procedure exists^26^, it has been shown that models benefit from using a batch of random SMILES (augmentation) during training and inference^27–30^. Recently, such augmentation was also applied to reaction modeling^11,18,31,32^. The augmented (also sometimes called “random”) SMILES are all valid structures with the exception that the starting atom and the direction of the graph enumerations are selected randomly.

In this article, we scrutinize the various augmentation regimes and show that augmentation leads to better performance compared to the standard beam search inference or evaluation of the model under different temperatures. We clearly mention that our study is to predict single-step and not multi-step retrosynthesis, which has been also targeted using transformer^33,34^. We show that by using more complicated data augmentation strategies we decrease overfitting^35^ of neural networks and increase their accuracy to achieve top performances for both direct and retro-synthesis. We observe that the harder are the data to train the model, the better it will predict new ones. Moreover, we introduce a new measure MaxFrag accuracy for the prediction of the largest fragment (thus identifying principal transformation for classical retro-synthesis).

## Results

The baseline dataset contained only canonical SMILES. The other datasets also contained SMILES, augmented as described in the section Supplementary methods. Four different scenarios were used to augment training set sequences. Namely, we used augmentation of products only (xN), augmentation of products and reactants/reagents (xNF), augmentation of products and reactants/reagents followed by shuffling of the order of reactant/reagents (xNS), and finally mixed forward/reverse reactions, where each retrosynthesis reaction from xNS was followed by the inverse (forward synthesis) reaction (xNM). Only the simplest augmentation xN was used for test sets because no information about reactant/reagents could be used for the retrosynthesis prediction. At least one copy of canonical SMILES for each reaction was present in all augmentation scenarios.

### Reaction synthesis data

We used a training set filtered from USPTO database^36^ containing 50 k reactions classified into 10 reaction types. We used splitting proposed by Liu et al.^12^ and divided it into 40, 5, and 5 k reactions for the training, validation, and test sets, respectively. As in the previous study^13^, after observing that early stopping using the validation set did not improve model test accuracy (the model performance for each of the sets was monotonically increasing with number of iterations, see Supplementary Fig. 1), we combined the training and the validation sets into a combined training set. The 5 k test reactions were predicted only once the model training was finished and were not used at any stage of the model development. In a similar way we joined training and validation sets of USPTO-MIT^22^ dataset for direct reaction prediction. In order to provide a more straightforward comparison with results of the previous studies we also reported performances when developing models using only the respective training sets. Moreover a model with the largest published USPTO-full set^24^ was also developed.

### Analysis of canonical datasets

The development of a model with canonical SMILES (x1) as the training set provided 40.9% accuracy for prediction of the canonical test set. An attempt to use this model to predict the augmented test set (x5, x10), resulted in much lower top-1 predictions of 23.3% and 18.4%, respectively. This result was to be expected, because the model trained with only canonical sequences was not able to generalize and predict augmented SMILES, which use different styles of molecular representation.

### Augmentation of products only (xN)

The augmentation of the products (input data), with just one additional augmented SMILES x2, increased top-1 accuracy to 43.7% for the test data composed of canonical sequences. Increasing the number of augmentations in the training set did not increase the top-1 prediction accuracy. Thus, the augmentation of the training set with just one random SMILES contributed the best performance. This result is in concordance with another study where only one random SMILES was used to augment data^18^.

### Analysis of character and exact sequence-based prediction accuracy

**Fig. 1:**
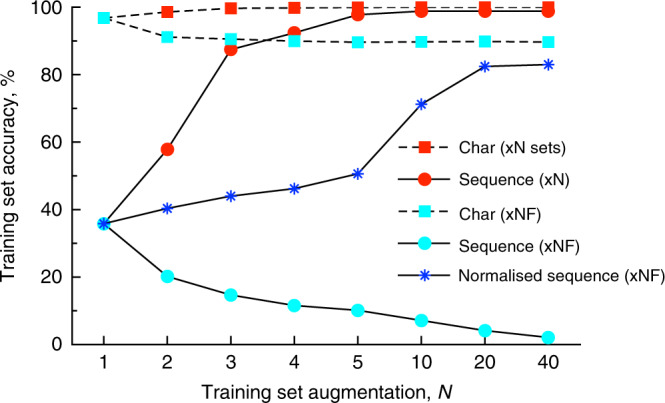
Character and exact sequence-based accuracies calculated for the monitoring set. The transformer memorized the target sequences if the target sequences were all canonical SMILES (red dots). It also reasonably predicted the sequence composition for randomized target SMILES (cyan rectangle, dashed) but its performance decreased for prediction of exact full SMILES (cyan circle). The performance normalized by the percentage of canonical sequences increased with the number of augmentations, *N*, since some of the random sequences were canonical ones.

To better understand the model training, we also developed several models where approximately 10% of the dataset did not participate in training but was used to monitor its prediction performance. Different from the test set, which tested the performance of models when predicting a new reaction, the monitoring set tested the ability of the Transformer to predict different SMILES generated for the same reaction. The Transformer was able to recognize different representations of the same reaction. For example, when training x1, the character and exact sequence-based accuracies when predicting the monitoring sequences were 96.5% and 34.5%, respectively. The final performance for the test set, 40.9%, was higher because some reaction products from the transformer provided noncanonical SMILES, which were correctly matched after transformation to canonical ones. When using augmented training sequences (x10), the accuracies increased to 99.97% and 98.9%, for character and exact sequence-based accuracy, respectively (see Fig. 1). The transformer recognized different representations of SMILES for reactants and reagents of the same training set reaction, and was able to exactly restore the target products which were memorized. Demonstrably, it was also able to memorize any random sequences. To show this, we used a random SMILES sequence (xNR set in Supplementary Tables 1 and 2, and Supplementary Fig. 2) instead of the canonical sequences as the target for prediction. While this task was more difficult and took more epochs to train, the transformer was able to perfectly memorize random sequences. Since the SMILES prediction target was random, the transformer was not able to learn canonicalization rules on how to write the target. Despite this fact, it still calculated a top-1 prediction accuracy of 26.8% for the test set which was, however, significantly lower compared to the 42.2% achieved using the x10 dataset with canonical sequences as the target.

### Augmentation of reactants and reagents

A boost of the Transformer performance was observed when, in addition to products, i.e., the inputs SMILES, we also augmented the target SMILES, i.e., reactants and reagents. This task was more difficult for the transformer, which resulted in a drop in both character and sequence based scores for monitoring sequences during the training stage. For example, when using the training dataset with one augmented SMILES, x2F, the character based accuracy dropped to 91.3%, which was lower than 98.6% calculated with the x2 dataset composed of canonical product SMILES (Fig. 1). For a larger number of augmentations, the character-based accuracy converged to a plateau, e.g., 89.96% and 89.67% for the x5F and x20F training sets, respectively. The character-based accuracy was calculated as the percentage of exact matches between target and predicted sequences, e.g., “CCCCN” and “NCCCC” have an accuracy of 80%, despite being the same SMILES but written from different starting atoms. Thus despite the fact that the Transformer faced a prediction of random SMILES, it was still able to provide a reasonable prediction of their character composition.

However, of course, the transformer was not able to predict the exact random product SMILES. This resulted in a decrease in sequence-based accuracy based on the number of augmentations for xNF training datasets (Fig. 1, cyan circle). Still the transformer was able to predict some of the sequences, which corresponded to the subset of canonical sequences in the monitoring set. Interestingly, the sequence accuracy normalized to the percentage of canonical SMILES in the monitoring sets increased with the number of augmentations since some randomly generated sequences were canonical SMILES.

### Top-1 performance analysis

**Fig. 2:**
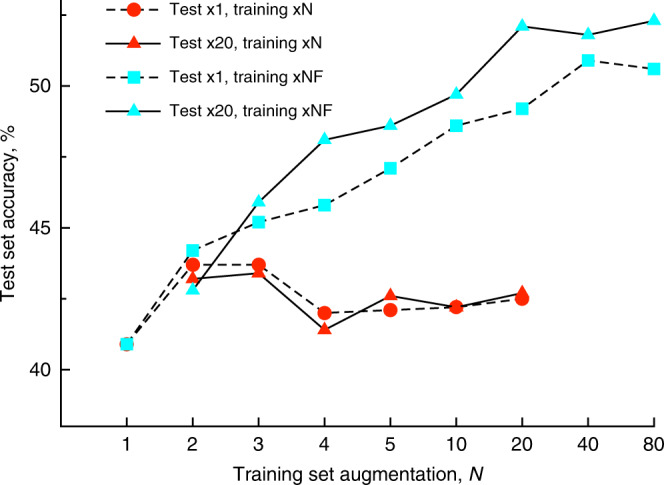
Top-1 performance of models developed with different number of augmentation (shown on *x*-axis) and different augmentation scenarios applied to both test and training sets (red color: only products were augmented; cyan color: full reactions were augmented). The use of the large number of augmentations for the test set (solid lines) improved prediction accuracy for models developed with augmentation of full reactions but did not influence the performance of models where only input data were augmented.

For augmentations with 1 or 2 random SMILES, the top-1 prediction performance of the models trained with augmentation of reactants and reagents only, xN, and full reaction augmentation, xNF, were similar. For a larger number of augmentations the models trained with xNF sets had systematically better performances than those developed with xN sets (Fig. 2). The training with the x80F set provided the highest top-1 performance of 52.3% when this model was applied to the test set generated with x20 number of augmented sequences. While it was possible that further increase in the augmentations could still increase the top-1 performance, we did not perform such calculations due to limitations on available computational resources.

### Shuffling order of reactants

In addition to augmenting the full reaction, we also randomly shuffled the orders of reactants (see xNS set description in Supplementary Tables 1 and 2). The effect of this additional data perturbation improved top-1 performance to 53.1% for the x20S training dataset applied to the test set with the same number of augmentations (Supplementary Fig. 3). Further increasing the number of augmentations resulted in the loss of top-1 prediction accuracy.

### Shuffling and mixing of retrosynthesis and direct reactions

The training of retrosynthesis and direct reactions simultaneously could create a mixed representation of latent space and further increase the ability of the transformer to make generalizations. We tested this hypothesis by combining direct and reverse reactions in one training set by reversing the order of product/reactant + reagents and adding a dot to distinguish direct reactions (see e.g., Supplementary Table 2, x2M augmentation). Contrary to previous analysis, which required 20 augmentations of training set sequences to achieve the highest performance, the mixed dataset achieved it with only 10 augmentations (Supplementary Fig. 3). Since the mixed dataset also included direct reactions, it had the same number of 19 augmented SMILES per canonical SMILES as in the previous analyses. Thus, this number of augmentations was optimal for the training of the transformer. A smaller number of augmentations did not allow it to fully explore the data diversity while a larger number created too much noise and made it difficult to learn canonization rules, which were injected by the single canonical sequence. For the x10M training set, the transformer calculated 52.8%, which was similar to 53.1% calculated using the x20S training dataset.

### Top-5 performance analysis

This measure provided a relaxed estimation of the performance of the model by measuring if the correct reaction is listed in the top-5 predicted reactions. Actually, it is questionable whether for retrosynthetic models having the highest top-1 accuracy is desirable. The goal of a retrosynthetic model is to obtain several precursor suggestions and not exclusively the ones stated in the literature. Moreover, multiple reactions for the same product exist. An example includes the aromatic substitution of an aryl halide (R-X) to an aryl amine (R-NH2) or aryl hydroxide (R-OH). Models with higher top-n scores do suggest other probable reactions (indeed, all reactions amid top-n have similar probability) which may correspond to those not reported in the literature for the analyzed example. Thus models with higher top-N scores but with similar top-1 scores could be interesting for a chemist since they do propose the correct prediction along with similar quality top-1 reactions.

**Fig. 3:**
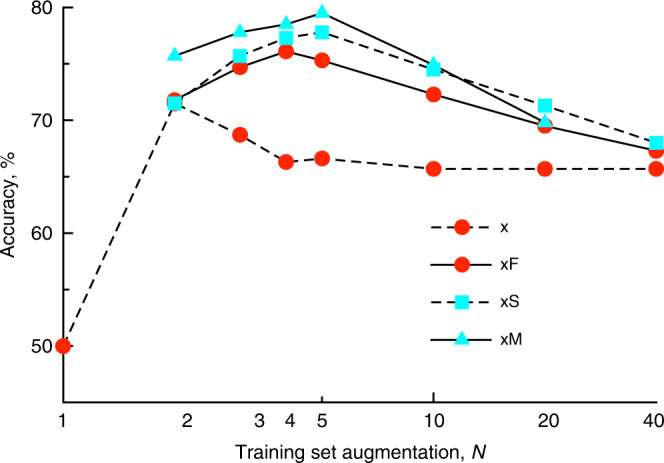
Top-5 performance of transformer models developed with different training set augmentation protocols for prediction of the x20 test set. Supplementary Tables 1 and 2 for description of the respective protocols.

For each number of augmentations, the top-5 performance generally increased with the number of augmented sequences. The highest top-5 value was consistently calculated across different scenarios for training sets with 4–5 augmentations only (Fig. 3). The highest accuracy, 78.9%, was calculated for the mixture dataset using the x5M training set augmentation. This number had approximately 1% higher accuracy than that calculated using the x5S training set (Fig. 3).

### Reference USPTO-50 k model

For all studies we used a fixed number of epochs *N* = 100. However, we needed to confirm that this was a sufficient number of epochs and to determine if we could calculate better results by training for longer. We selected the model developed with the x5M training set, which provided the highest performance for top-5 accuracy, and trained it for an additional 400 iterations (in total 500). This additional training improved top-1 accuracy to 53.3% while top-5 performance increased to 79.4% (Table 1) when using beam = 5, e.g., the same as in previous analyses.

**Table 1:** Analysis of the reference model performance depending on the parameters of the application protocol.

	Test set x1		Test set x20		Test set x100		
Apply model setting	Top-1	Top-5	Top-1	Top-5	Top-1	Top-5	Top-10
Reference accuracy^a^	48.5	72.5	53.3	79.4	53.6	80.8	85
Temperature, *t* = 1.3	49.1	67.7	52.7	77.7	53.3	78.4	83.2
No beam search, i.e., beam = 1	47.7	47.7	53.3	75.3	53.8*	78.8	81.7
Beam size, beam = 10	48.3	73.4	53.5	80	53.5	81*	85.7
Beam size, beam = 44	48.3	72.5	53.5	80	53.5	80.5	85.8*

^a^The final reference model was built using 500 iterations for the x5M training set. Its reference performance was evaluated using beam size = 5, temperature = 1. The altered parameters are shown for several other application scenarios. For beam = 1 and x1000 augmentations the model calculated 53.7, 80 and 84.3 for top-1, top-5, and top-10 predictions, respectively. This augmentation as well as the one with beam size = 10 applied to x100 analyzed the same number of predicted sequences.

* Indicate the best results. Larger beam sizes contributed better results for larger top-*n* predictions.

Further improvement was achieved by using a large number of augmentations, and x100 as the test set. With this setting the model achieved an accuracy of 53.6% and 80.8% for top-1 and top-5 predictions, respectively.

### Influence of temperature

In our previous study^13^, we observed that using higher temperatures during beam search increased model accuracy for the top-1 prediction. It should be mentioned that no augmentation was used in that study. Under the same experimental setup with no augmentation, i.e., when predicting test set composed of only canonical sequences, x1, the top-1 accuracy of the model increased from 48.3% to 49.1% and 49.2% when using temperatures 1.3 or 1.5, respectively. However, the top-1 and top-5 performances for the augmented data (x20) decreased from 53.3% to 52.7% and 52.4%, respectively. For the same test set the top-5 accuracies also decreased from 79.4% to 77.7% and 77.4% for both temperatures, respectively. Thus, while higher temperatures increased the variability of predictions and thus performance for prediction of canonical sequences, its effect was negative for the augmented data. In particular, it resulted in the lower accuracy of top-5 predictions.

### Influence of beam search

In the above studies we consistently used a beam size of 5 for all analyses. The goal of the beam search was to generate multiple predictions for the same data and thus to better explore the variability of predictions. For example, when using the x20 test set and a beam size of 5, we obtained up to 100 individual predictions, which were used to select the most frequently appearing top-1 and top-5 sequences. Increasing the beam size to 10 further improved top-1 by 0.2 to 53.5% and top-5 by 0.6% to 80% for the test set. The decrease of the beam size to 3 provided a slightly higher top-1 score of 53.4% but decreased the top-5 to 78.5% for the same test set. The use of beam size 1 resulted in a top-1 accuracy of 53.3% and a reduced top-5 accuracy of 75.3% (Table 1). These results were expected: the variation of the beam size slightly influenced the identification of the highest ranked sequence but its smaller number reduced exploration of the space of other top-reactions for larger *n*.

Both beam search and augmentation increased the number of predicted SMILES which in turn led to better accuracy of model predictions. Thus both of these methods could contribute to the generation of multiple predictions to be used to identify top-ranked sequences. The maximum size of the beam was restricted by the size of the target vocabulary (number of characters in the target SMILES), which was 44 characters for our dataset. Because of the design of the beam search and because we explicitly excluded duplicated predictions (see section “Analysis of predicted SMILES” as well as Supplementary Table 3), the dataset used for analysis did not generate duplicated sequences for the same beam search. However, such sequences were indeed generated at different positions of the beam as different representations of the same SMILES. The number of non-unique sequences generated within the same beam search increased with the length of the beam. Interestingly, the use of canonical SMILES as input data contributed to the largest number of unique SMILES, which were 86%, 82% and 78% for beam searches of size 5, 10, and 44, respectively. The use of augmented random SMILES as input contributed smaller numbers of unique sequences, e.g., 42%, 28% and 13% for beam searches of size 5, 10, and 44, respectively. For both types of SMILES some generated SMILES were erroneous and could not be correctly converted by RDKit. Such sequences were excluded from analysis. For large beam sizes, canonical SMILES produced a much bigger percentage of incorrect SMILES, as compared to the use of random SMILES (see Supplementary Fig. 4). The large difference in the results generated when starting from canonical and random SMILES was also observed for analysis of the percentage of correct predictions for each beam position. In general, the number of erroneous SMILES was low, e.g., on average it was less than 1% and 3% for beam search 10, when using augmented and canonical SMILES as input, respectively (Supplementary Fig. 4). While graph-based methods predict exact chemical structures and thus have 0% syntactically invalid SMILES, a few percentage points of incorrectly predicted structures by the transformer model does not make a large difference to these methods.

The use of canonical SMILES provided (Supplementary Fig. 4) a higher accuracy for the first beam position, but its accuracy was much lower for other beams. This was because the Transformer generated canonical SMILES for the canonical input sequences (e.g., 91% of valid SMILES produced at the position 1 of the beam search for input canonical SMILES were canonical ones) and since only one valid canonical SMILES could be produced, it failed to generate new correct SMILES. Indeed, during the training phase, the transformer always had a pair of canonical SMILES as input and target sequences. Contrary to that, using augmented SMILES allowed more freedom and allowed it to contribute valid but not necessarily canonical SMILES (e.g., only 33% of generated SMILES at the position one of the beam search were canonical ones if augmented SMILES were used as input).

The decrease in performance of SMILES generated when using canonical SMILES was one of the main reasons to implement deduplication of data and retain only the first SMILES for the prediction of reactions (see section “Analysis of predicted SMILES”). When deduplication was not performed and all SMILES generated during the beam search were used to rank predictions (compare Supplementary Tables 3 and 4), the top-1 performances of models were most significantly affected when using only few augmentations, e.g., for the reference model its accuracy dropped from 48.3% (reference prediction, Table 1) to 47% but did not change for, e.g., top-5 performance. In principle, the analysis retaining multiple predicted sequences was based on more data and thus was more stable. Therefore, it could be used when several augmentations and/or large values of top-*n* are used for analysis.

As it was mentioned above, both data augmentation and beam search could be used to generate multiple predictions. For the same number of generated sequences, 1000 per SMILES, using a beam = 10 search for the x100 set produced lower accuracy, 53.5% compared to 53.7% using augmented data with the x1000 test set without any beam search. The performance of both methods were the same and equal to 53.7% when the deduplication procedure was not used. However, the beam search contributed to better accuracy, i.e., 81% vs. 80% and 85.7% vs. 84.3% compared to the use of augmententation alone for top-5 and top-10, respectively. Thus, using beam search allowed a better exploration of data when suggesting several alternative reactions. In any case the augmentation was a very important part of the beam search and for the best performance, both of these approaches should be used simultaneously. We also do not exclude that optimization of the augmentation may improve its results in the future. Moreover, data augmentation used alone without a beam search contributed superior models to the beam search used without any data augmentation.

### Accuracy of prediction

**Fig. 4:**
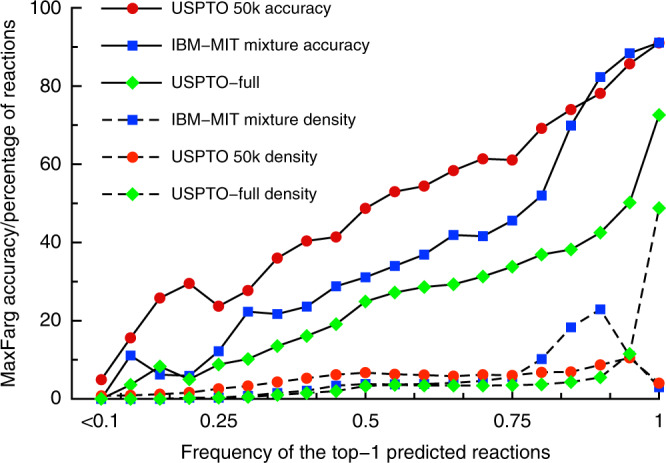
Accuracy and density (fraction of predictions) of the Transformer for MaxFrag top-1 retrosynthesis accuracy. The accuracy and density are shown as a function of the frequency of appearance of the top-1 SMILES in the output of the Transformer for the respective test sets of the models.

For some reaction predictions without the use of augmented sequences or position at the beam search the majority of predicted sequences were identical, while for other reactions the Transformer generated as many different SMILES as possible reactants (see Supplementary Table 5). While the beam generation procedure guaranteed that each prediction had exactly the same sequence of characters, in many cases the Transformer produced multiple noncanonical instances of the same SMILES. The frequency of the appearance of the most frequent (after conversion to the canonical representation) SMILES could, therefore, indicate the confidence of the transformer in the prediction. Figure 4 indicates such frequency (which was calculated on 100x augmented dataset) correlated well with the accuracy of prediction and could be used as a confidence score for the chemist. Indeed, the reactions in which the most frequent SMILES dominated amid all predictions for Top-1 were likely to be predicted correctly. If the most frequent SMILES had low frequencies, such predictions were likely to be incorrect ones. For about 20% of the most frequent predictions, the accuracy of the retrosynthesis prediction was above 80% and for 4% more than 90%. It should be mentioned, that for a practical implementation which critically depends on the speed, e.g., multistep synthesis, there is no reason to always run all 100 predictions to get the confidence estimations. One can always estimate the probability of the most frequent SMILES and its confidence interval based on a much smaller number of predictions thus decreasing the number of calculations.

As shown in Fig. 4, the same correlations were observed for two other datasets USPTO-MIT and USPTO-Full, which are analyzed in the following section. The same approach can be used for top-*n* predictions by suggesting one or more plausible pathways for retrosynthesis. An example of such correlations for Top-5 MaxFrag accuracy were shown in Supplementary Fig. 5. Moreover, the same approach also predicted the accuracy for the direct synthesis as it was demonstrated at Supplementary Fig. 6. It should be mentioned that use of data augmentation is not the only approach to estimate the accuracy of predictions, and other methods based on the likelihood of the direct reaction prediction were also proposed^18,34^ and were shown to correlate with the accuracy of the predictions. A comparison of such methods is beyond the scope of this study.

### Analysis of prediction accuracy for different scenarios

The accuracy of the reference model was about 5% to 7% (top-1) and 10% (top-5) higher for reactions without stereochemistry than for those with it (Table 2). 20% of the reactions in the test set contained molecules with stereochemistry. An increase in the number of augmentations of the test set increased the accuracy of both stereo and non-stereochemical reactions. Stereochemical reactions in the dataset may also suffer from a larger number of annotation errors or/and can have lower prediction scores since such data were underrepresented in the training set. In addition, for some reactions despite the relative stereochemistry being conserved it may still define confusing information for the model due to the reactant satellite effect. The R/S could be also affected by the way the SMILES was written, e.g., from A to Z or Z to A.

**Table 2:** Prediction accuracy of the reference model for different subsets of the test set of USPTO-50k using a beam search of size 10.

	Top-1			Top-5			Top-10		
Test set augmentation	All	Stereo (20%)	No stereo (80%)	All	Stereo (20%)	No stereo (80%)	All	Stereo (20%)	No stereo (80%)
x1	48.3	44.7	49.2	73.4	67.3	74.9	77.4	71	79
x20	53.4	47.3	55	80	73.3	81.9	84.2	79.2	85.4
x100	53.5*	47.1	55.1	81*	74.6	82.6	85.7*	81.2	86.8
MaxFrag,^a^ x1	53.5	48.7	54.7	79.2	72.7	80.9	81.6	75.1	83.3
MaxFrag, x20	58.5	52	60.1	84.7	79	86.1	88.6	83.6	89.8
MaxFrag, x100	58.5*	51.2	60.3	85.4*	79.4	86.9	90*	85.1	91.2

^a^The classical retro-synthesis accuracy was estimated as the percentage of correctly predicted largest fragments, i.e., “maximum fragment” (MaxFrag) accuracy.

* Indicate the best results.

### Classical retro-synthesis accuracy: recognition accuracy for the largest fragment

**Fig. 5:**
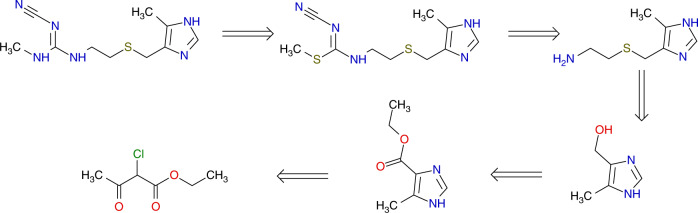
Classical representation of the retrosynthesis of cimetidine focusing on the principal transformations, as is typically written by synthetic chemists (adapted from https://de.wikipedia.org/wiki/Cimetidin under CC BY-SA 3.0 license). The currently used top-n accuracy measures also include prediction of other reactants^12,13,19,20.24,32^, which may not be necessary for classical retrosynthesis planning.

The prediction of SMILES for retro-synthesis includes exact prediction of the reactants. However, the same reaction performed using different reactants can result in a similar yield. In general the database does not contain all possible reaction conditions to make a given product. Therefore, a prediction of only the main (the largest) reactant can be considered more relevant for retro-synthesis predictions, since we need to first identify the reaction type. Indeed, a chemist generally writes a retrosynthesis by decomposing a target molecule into pieces. This classical procedure, focusing only on main compound transformations, is the minimal information required to get an efficient retrosynthesis route and at the same time all reactions needed (see Fig. 5). The selection of reaction conditions of the reactions can be considered as a subsequent task.

That is why we decided to consider the recognition of the largest reactant as a new measure of the model performance: classical retro-synthesis accuracy, i.e., the accuracy of prediction of the “Maximal Fragment” (MaxFrag). The MaxFrag was 85.4% for the top-5 reaction prediction (Table 2). The MaxFrag is important to estimate an ability of a system to automatically deduce the correct reaction class. This strategy is orthogonal to explicitly providing reaction class information as input to a model^24^. Adding the reaction class as prior information is equivalent to getting a hint on an exam: this is impractical and also reduces the chance of proposing alternate feasible reactions. Using MaxFrag is more accurate and logical than providing a reaction class as prior information. Besides MaxFrag and Top-*n*, other scores were proposed to evaluate the success of retro suggestions/reactions, e.g., the matching score by Satoh and Funatsu^37^, the “in-scope” filter by Segler et al.^5^, and the forward transformer score by Schwaller et al.^34^. However, MaxFrag is the easiest and the interpretable one.

### Retrosynthesis data quality and MaxFrag accuracy

The use of the classical retro-synthesis accuracy (MaxFrag top-*n*) calculated a systematic higher accuracy in comparison to the traditional top-*n* scores. To explain this fact, we analyzed our datasets and found four types of reactions: non-reagent reactions, one reagent reactions, multiple reagent reactions, and unclear reagent reactions. Non-reagent reactions were reactions that did not work (i.e., A + B− > A). One reagent reactions had only one starting material for the product (A− > P), multiple reagents had multiple starting materials for the same end product (A + B− > P), and finally unclear reagents where the reaction conditions, solvent, salts, and so on, were included as reagents (A + B + N− > P, where N were chemicals that did not participate to form the product). Depending on the dataset the proportions of these reaction categories slightly varied. In the MIT dataset around 0.5% of reactions were non-reagent reactions, and around 10% of the reactions were unclear reagent reactions while there were less than 1% of such reactions in the USPTO-50 k dataset. Thus, for the MIT set it would be impossible to fully predict about 10% of reactions for the retrosynthesis, since they contained chemicals “N” that did not form the reaction, but only conditions, solvent, etc. This more challenging problem of predicting not only the reactants but also the reagents, while still keeping diverse precursor suggestions was addressed elsewhere^34^. For the direct synthesis that was not a severe problem since the transformer could correctly identify and pick-up the interacting components (“A” and “B”) and predict the product. However, the use of Top-*n* for retrosynthesis is questionable due to the aforementioned problem. The use of MaxFrag accuracy decreased those effects by focusing on the main reagent. That is why, in our opinion, the MaxFrag score better reflected the chemistry than Top-1 accuracy.

Still there is an unsolved challenge with this score due to the possibilities to synthesize the same products starting from multiple reagents. Both Top-*n* and MaxFrag Top-*n* scores were calculated by using the exact match of the predicted and target molecules. But, for example, in the reaction R-R1 + NH3− > R-NH2 multiples choices of R1, i.e., -OH, -Cl, -Br, -I, or -F, would be all correct predictions. The only difference would be the reaction rates and yields, which are not part of the prediction algorithms. Unfortunately the currently used scores, including MaxFrag, cannot yet correctly account for this problem. The problem to some extent could be alleviated by using Top/MaxFrag-*n* instead of Top/MaxFrag-1 scores: by considering multiple reagents generated by the model, we could also get the one provided in the initial reaction. Thus, the retrosynthesis task is not about getting high Top-1 accuracy. Any classical organic synthesis book, such as the famous Larock’s “Comprehensive Organic Transformations”^38^ indicates multiple ways to synthesize chemical compounds and this has to be reflected in the score. The classical retro-synthesis accuracy measured by MaxFrag is a first attempt to better handle those data ambiguities during the validation process and we highly encourage other users to use it. However, in order to enable a comparison with the previous studies we also reported traditional Top-*n* scores.

### Analysis of prediction accuracy for different classes

**Fig. 6:**
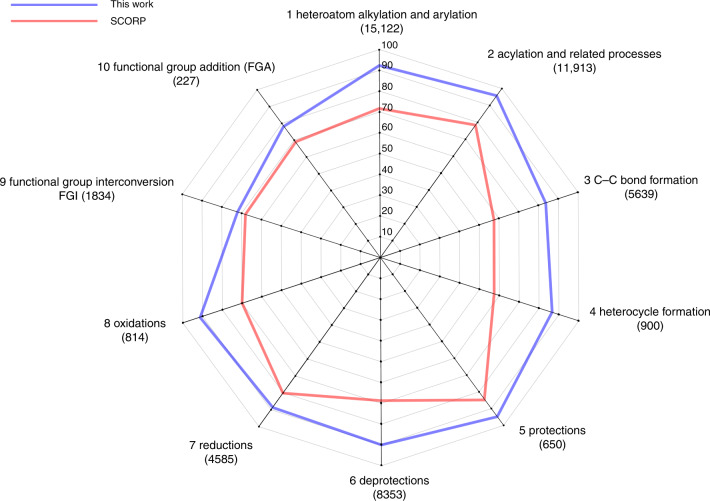
Top-10 accuracy of prediction of different classes of reactions.

The original dataset USPTO-50 k set^12^ provides a reaction type label for every reaction. In total, ten reaction classes ranging from protection/deprotection, to carbon–carbon bond and heterocycle formation present the most common reactions in organic synthesis. The comparison of accuracy for each class of reactions was presented in Fig. 6. Our best model showed excellent results, outperforming the state-of-the-art Self-Corrected Transformer^19^. Functional group interconversion and addition, as well as carbon–carbon bond formation were the most difficult for the models to predict. It was not surprising, due to the diverse possibilities for choosing reactions and corresponding reactants for C–C bond creation compared to more straightforward oxidation or protection where the set of groups and reactants is more narrow.

### Prediction of direct reactions

The same strategy described in this work was applied to predicting direct reactions from the USPTO-MIT dataset^22^. We used 439 k reactions (training and validation set were joined together) as the training set and predicted 40 k reactions from the test set by training the transformer with the same architecture and parameters. The separated and mixed sets were used. In the separated set reactants and reagents were separated with the “>” sign while in mixed set all “>” are substituted with “.” and the order of reactants and reagents was additionally shuffled. The mixed set was more difficult for training since the transformer had to identify the reaction center from a larger number of molecules. However, such a set better reflected a practical application since separation of data on reactants and reagents in some cases would not be possible without a knowledge of the target product, and thus it did provide a hint to the transformer about the reaction center. We have removed 316 reactions from the training set where the largest products had length smaller than five characters (no reactions were removed from the test set). The Transformer was training using the x5N augmentation protocol for the separated set as well as the x5S and x5M protocols for the mixed set. Since it would be impractical to predict all reagents and reactants for the retrosynthesis task, which was used to additionally augment data in the x5M protocol, only the largest reactant was retained as a target for the reverse reactions. Augmented test sets were predicted using beam size 10 (Table 3). For the mixed test set the order of reactants and reagents was shuffled.

**Table 3:** Prediction accuracy for direct reaction from USPTO-MIT test set using beam size = 10.

	Test set x1			Test set x20			Test set x100		
Training set	Top-1	Top-5	Top-10	Top-1	Top-5	Top-10	Top-1	Top-5	Top-10
x5N (separated)	91.1	96.3	96.7	91.8	96.9	97.3	91.9	97	97.4
x5S (mixed)	90	95.8	96.2	90.4	96.4	96.9	90.4	96.5	97
x5M (mixed)	90	95.5	95.7	90.2	96.1	96.5	90.2	96.2	96.8

As in previous studies, separation of reagent and reactants with “>” symbols contributed to a model (x5N) with higher prediction scores than for models with mixed sets (x5S and x5M). The additional augmentation of data using retrosynthesis reactions (x5M) did not improve the model. This could be due to the fact that the data for direct reactions were much larger and already contained sufficient information to develop accurate models. While using the x100 test set still contributed better prediction accuracy than using x20, the improvements were in the order of 0.1% or no improvement at all. Thus the effect of using larger augmentations on model performance reached saturation for the x100 test set.

### Comparison with published models for direct synthesis using USPTO-MIT set

The USPTO-MIT was used as benchmarking for direct synthesis predictions in multiple articles. The AT provided the highest gain in performance for prediction of the more challenging mixed dataset (Table 4). Since the model was trained with randomly shuffled augmented data, it was able to generalize very well and provide excellent predictions for the new mixed data. In order to provide a more adequate comparison with previous studies we also developed a model based on exactly the same training data of 400 k. Interestingly, the use of a smaller dataset slightly increased Top-1 performance to 90.6% but decreased Top-5 performance to 96.1. It should be noted that improvements for direct synthesis look small, i.e., just few percentages. Indeed, the model performance for the direct synthesis increased from 88.6 to 90.6 (Top-1) and 96.1 from 94.2 (Top-5) as compared to the single model reported in ref. ^18^. Actually, this is a significant increase in performance since AT decreased the relative errors by 15% and 30% for both sets, respectively, if we consider that we can predict direct synthesis with 100%. In reality we approach the experimental accuracy and further decrease of the errors will be challenging.

**Table 4:** Comparison of recently published methods for direct synthesis prediction on the USPTO-MIT set.

Model	Top-1		Top-2		Top-5		
	Separated	Mixed	Separated	Mixed	Separated	Mixed	Ref. #
Transformer (single model)	90.4	88.6	93.7	92.4	95.3	94.2	^18^
Transformer (ensemble of models)	91		94.3		95.8		^18^
Seq2Seq	80.3				87.5		^11^
WLDN	79.6				89.2		^32^
GTPN	83.2				86.5		^40^
WLDN5	85.6				93.4		^23^
AT, this work^a^	91.9	90.4	95.4	94.6	97	96.5	
AT trained with same training set as in ref. ^22^.	92	90.6	95.4	94.4	97	96.1	

^a^The results of the models applied to x100 augmented dataset using beam size = 10. Model was trained on a set of 439 k reactions, which combines both the training set of 400 k and the validation set of 39 k from ref. ^22^. The model was trained on the 400 k training set to better match performance of previous models.

### Comparison with published models for retrosynthesis tasks

#### USPTO-50 k

The proposed augmentation protocol achieved the best published results on the USPTO-50 k dataset (Table 5). In the previous studies with this set the authors separated data on training, validation and test sets. In all our analyses, since the validation set was not used for model selection and we did not observe the model overfitting^35^, we joined training and validation sets to use all data in order to develop better models. While we think this is a fair comparison (it is up to the developers of the model to decide on how to best use the available data), we also added results when the model was developed with only the 40 k compounds for USPTO-50 k set (Table 5). The accuracies of the models developed with 40 and 45 k sets were very similar for the test set. Thus, the data augmentation allowed to compensate for the smaller size of the training set.

**Table 5:** Comparison of retrosynthesis recently published methods for retrosynthesis prediction on USPTO-50 k.

Model	Top-1	Top-2	Top-5	Top-10	Ref. #	Comments
Seq2Seq	37.4		57.0	61.7	^12^	40/5/5 split; splitting any reactions with multiple products into multiple single product and removal of trivial products
Transformer (3*6)	42.7	52.5	69.8	–	^13^	45/5 split: no validation set was used
Transformer (6*8), (self corrected)	43.7		65.2	68.7	^19^	40/5/5 split, reagents from reactants are removed
Transformer, augmentation	44.8	57.1	57.7	79.4	^32^	Same as in ref. ^12^.
Similarity-based	37.3		63.3	74.1	^20^	Same as in ref. ^12^.
Graph Logic Network	52.5		75.6	83.7	^24^	Same as in refs. ^12,19^.
G2Gs	48.9		72.5	75.5	^25^	Same as in ref. ^12^.
AT^a^	53.5	69.4	81	85.7		Same as in ref. ^13^.
AT	53.2	68.1	80.5	85.2		Only 40 k samples were used as training set to match the other results
AT MaxFrag^b^	58.5	73	85.4	90		Same as in ref. ^13^.
AT MaxFrag	58	73.4	84.8	89.1		Only 40 k samples were used as training set to match the other results

^a^The results of the reference model applied to x100 augmented dataset using beam size = 10.

^b^The classical retro-synthesis accuracy was estimated as accuracy for prediction of the largest fragment (MaxFrag).

#### USPTO-MIT

We also analyzed the performance of the model at retrosynthesis of the USPTO-MIT set. Compared to USPTO-50 k this database also contained multiple reagents and possible catalysts. In our previous analysis (Table 3) we used the retrosynthesis of the largest fragment as part of the “mix” protocol (x5M), i.e., the products were used as input data contained with “.” to predict the largest reactant (as explained in the Supplementary materials, in order to distinguish both direct and retrosynthesis reactions, one of them started with a dot). The dot in front of the SMILES allowed the Transformer to distinguish retrosynthesis from the primary studied direct synthesis reaction. But, of course, the model trained with such data could be also used for retrosynthesis, provided that input data also started with “.”. We also developed a new retrosynthesis model for this set by making it more compatible to USPTO-50 k. For this we kept only the 1–2 largest fragments as the targets for retrosynthesis prediction and trained a new model using the x5S protocol. Both models were used to predict the 40 k test set which was augmented 100 times. The MaxFrag performances of x5S model, 61.9% (Top-1), 84.4% (Top-5), and 86.9% (Top-10) were very similar to those calculated for the USPTO-50 k set (58.5, 85.4, and 90—see Table 5). The x5M model, which as aforementioned was a “by-product” of our direct reaction predictions, calculated MaxFrag of 61.1%, 84.4% and 88.2% for the MaxFrag Top1-,Top-5, and Top-10, respectively. Considering that the USPTO-MIT set contained more diverse reactions than USPTO-50 k, this result clearly demonstrated the excellent performance of the developed approach and its scalability. The augmented transformer (AT) was able to improve its performance for the Top-1 by extracting knowledge from a much larger dataset of reactions.

#### USPTO-full

The final testing was done using a USPTO-full set by Dai et al.^24^. The authors created a large dataset from the entire set of reactions from USPTO 1976-2016. For reactions with multiple products they duplicated them into multiple ones with one product each. The authors also removed duplications in reactions as well as those with wrong mapping to obtain train/valid/test datasets with 800/100/100 k sizes. Our analysis identified that some reactions in these sets were still invalid, e.g., contained no products or just single ions as reactants (e.g., US08163899B2,>>[OH2:11]; US06048982,CC(=O)OCCCCC[I:22]>>[I-:22]; US07425593B2,>>[K:12]; US08114877B2,CC[I:13]>>[I-:13]). We eliminated such reactions as well as those where reactants had less than five atoms in total, since these were unlikely to be correct reactions. This procedure decreased sizes of the train/valid/test sets on average by 4% to 769/96/96 k. The AT trained using x5M protocol using the 769 k training set calculated the higher performance compared to results from the previous study (Table 6). Considering that after the removal of the 4% erroneous reactions the test dataset was decreased, we also included recalculated performance for it by assuming the worst case scenario: that AT and other tested methods failed for all excluded sequences. Even for this very conservative estimation the AT provided significant improvements compared to previously reported results. The MaxFrag accuracies for USPTO-full were lower compared to that of other analyzed sets due to the much higher diversity of this set.

**Table 6:** Top-k accuracy for retrosynthesis prediction on USPTO-full dataset.

	Retrosim^20^	Neuralsym^3^	GLN^24^	AT^a^
Top-1	32.8	35.8	39.3	46.2 (44.4)^b^
Top-2				57.2 (54.9)
Top-10	56.1	60.8	63.7	73.3 (70.4)
MaxFrag top-1				54
MaxFrag top-2				66.3
MaxFrag top-5				77.3
MaxFrag top-10				80.6

^a^Model was trained using a filtered training set of 769 k from ref. ^20^.

^b^Accuracies in parentheses correspond to those recalculated for the test set by assuming that AT failed for all 4% of excluded reactions. Results for retrosim and neuralsym approaches as reported by Dai et al.^24^.

Thus for all analyzed data sets the AT provided an outstanding performance by consistently and significantly overperforming all previously published models for all statistical performances.

## Discussion

This study showed that careful design of the training set was of paramount importance for the performance of the Transformer. Training the model to learn different representations of the same reaction by distorting the initial canonical data eliminated the effect of memorization and increased the generalization performance of models. These ideas are intensively used, e.g., for image recognition^39^, and have been already successfully used in the context of several chemical problems^27–30^, including reaction predictions^18,31^, but were limited to the input data. For the first time we showed that application of augmentation to the target data significantly boosts the quality of the reaction prediction. We also showed for the first time that the frequency of predicted SMILES could be used as a confidence metric for (retro)synthesis prediction and can provide quantitative estimation of the most probable reactions amid top-n predicted outcomes. It is very critical to estimate the quality of the reaction prediction since it could help to better prioritize multi-step retrosynthesis. The developed methodology is unique to the use of augmentation techniques, currently unavailable to GCNs^24^, which directly operates with graphs. The estimated accuracy of prediction can help to distinguish reactions, which are difficult to predict, from typo and erroneous reaction data, which will be important to clean up the reaction data and further improve model quality. We also introduced a new MaxFrag measure, classical retro-synthesis accuracy, which in our opinion better reflects the requirements for retrosynthesis analysis.

It should be mentioned that use augmentation was first studied by authors of ref. ^18^, who introduced transformer to chemistry and applied it to chemical reactions by using SMILES instead of the text sequences. The augmentation of input data, which was done in that article, provided only a minor improvement of their models. Because of its small impact it was not followed in several other Transformer-based works, including our own study^13,19^. In this article we brought an original idea on how to augment chemical data, which provided a significant improvement of the results for all analyzed datasets.

SMILES random augmentation had the ability to stabilize the model’s learning by adding more data and adding more randomness and freedom into the network. Remarkably, this augmentation functioned similarly to ensemble learning, allowing for better statistics and improving the performance of the model. Beam search and augmentation were complementary and our reference model in essence got better results than models developed using graph representation of molecules^24^, for which the use of similar data augmentation technique is currently not possible.

## Supplementary information

Supplementary Information

## Data Availability

Data and predictions that support the results of this study are available at https://github.com/bigchem/synthesis.

## Code availability

Source code to perform the data augmentation is available at https://github.com/bigchem/synthesis.
