# Supplementary material for: State-of-the-art augmented NLP transformer models for direct and single-step retrosynthesis
Source: Nat Commun. 2020 Nov 4;11:5575. doi: 10.1038/s41467-020-19266-y (PMC7643129; doi:10.1038/s41467-020-19266-y)
Supplement: Supplementary file 1 — Supplementary Information [file 41467_2020_19266_MOESM1_ESM.pdf]

State-of-the-Art Augmented NLP Transformer models for direct and single-step retrosynthesis

Igor V. Tetko[1,2], Pavel Karpov[1,2], Ruud Van Deursen[3], Guillaume Godin[3]

[1]Institute of Structural Biology, Helmholtz Zentrum München - Research Center for Environmental Health (GmbH), Ingolstädter Landstraße 1, D-85764 Neuherberg, Germany,
[2]BIGCHEM GmbH, Valerystr. 49, D-85716 Unterschleißheim, Germany
i.tetko and pavel.karpov"@" helmholtz-muenchen.de
[3]Firmenich International SA, D-Lab by Firmenich, Rue de la Bergère 7, CH-1242 Meyrin-Satigny, Switzerland
guillaume.godin@firmenich.com

Corresponding author: i.tetko@helmholtz-muenchen.de

Supplementary materials

Supplementary Methods

Model architecture

Following our previous study[1] we used the Transformer[2] architecture to train all the models. The key component of the Transformer architecture is a self-attention block equipped with internal memory and attention. During the training phase the block extracts and structures the incoming data, splitting it into memory keys and associated values. Thus, the block resembles a library, where all the books (values) are referred to by an index (keys). On a new request the model calculates the attention to the known keys and then extracts knowledge from the values proportionally. The Transformer shows excellent results not only on (retro) synthesis[1,3,4] tasks but also on ordinary classification and regression QSAR studies.[5]

The performance of the Transformer was estimated for the prediction of the whole training set after each epoch. The five models with the highest fraction of correctly predicted training set SMILES were stored. As a rule, the stored models correspond to the latest epochs of training. The weights of five stored models were averaged to form the final model, which was used to predict reactions from the test sets.

After several trials, we decided to use a Transformer architecture with 6 layers and 8 heads (6x8), which was used in the original work.[3] We found that using a smaller architecture with 3 layers and 8 heads (3x8), which was used in our previous study,[1] required more epochs to converge and thus longer overall training time to achieve the same performance. We restricted training of the model to 100 epochs to perform model development in a reasonable time and preserve the possibility to compare different augmentation approaches. For the final optimal architectures, we further investigated the effect of training time.

Influence of the batch size

The speed of calculations using augmented data was linearly increasing with the dataset size. One epoch using the USPTO-50k set (40k reactions) took 82s on a Titan V. Training of the USPTO-full augmented set (4.3M reactions) took 9514s, i.e. approximately 120 times longer. The use of a larger batch size (in our work we formed batches of length *ca.* 3000 characters, which approximately corresponded to 12-15 reactions and required about 3.5G of GPU memory for the given Transformer configuration) could increase the speed of calculations. However, we noticed that large batches (i.e., we tried a batch of length 30,000 characters on Tesla V100 with 32GB of memory) could result in a decrease in the speed of convergence. Therefore, for this study we used a batch with 3000 characters.

Beam search

When generating new SMILES, the Transformer predicted at each step probabilities for all characters from its vocabulary. There are two common approaches

to decoding from a linguistic model, such as a Transformer. The first one, a greedy search, always takes the element (symbol, word) with the maximum probability at each step. The second one, beam search,[6] tracks in parallel several possible decodings (beam size) and sorts them according to the sums of logarithms of probabilities of each element. Thus, beam search can select those decodings where at one step the element to be chosen has no maximum probability but later symbols have maximum so the overall sum is greater than in greedy search settings. The beam search with n=5 or n=10 beams were used to predict the test set for the majority of analyses performed in this study. As a result of a search using a beam with size n, the Transformer produced up to n SMILES. Because of the generation procedure these were always unique sequences. Some of them, however, could be errors or could be different representations of the same SMILES.

Augmentation

The datasets used in this study comprised both canonical and so-called augmented SMILES. Both canonical and augmented SMILES were generated using RDKit [7]. We introduced this SMILES free augmentation method into RDKit at the end of 2018 [8,9]. The augmented SMILES were all valid structures with an exception that the starting atom and the direction of graph enumerations were selected by chance. The augmentation increased the diversity of the training set.

The baseline dataset contained only canonical SMILES. The other datasets also contained SMILES augmented as summarized. Four different scenarios were used to augment training set sequences. Sequences were augmented using increasingly complex datasets as shown in Supplementary Tables 1 and 2. Namely, we used augmentation of products only (xN), augmentation of products and reactant/reagents (xNF), augmentation of products and reactants/reagents followed by shuffling of the order of reactant/reagents (xNS), and finally mixed forward/reverse reactions, where each retrosynthesis reaction from xNS was followed by the inverse (forward synthesis)

reaction (xNM). One more analysis was performed where the Transformer was asked to predict a fixed random SMILES string.

Only xN were used for augmentations of the test sets because no information about reactant/reagents could be used for the retrosynthesis prediction.

Supplementary Table 1. Augmentations of analyzed training datasets

**Dataset**	**Description**
xN	For N=1 the dataset contains canonical SMILES for reactants/reagents and products. For N>1 in addition to one canonical SMILES, the dataset contains (N-1) instances of the same reaction with augmented SMILES for the products (input data). The SMILES of reactants and reagents were canonical.
xNR	Products are encoded as canonical SMILES, but for reactants/reagents only one of possible random SMILES was chosen.
xNF	The first instances of each reaction contained canonical SMILES while other (N-1) instances were augmented for both input (products) and output (reactants and reagents) data. The order of SMILES in output data was not changed.
xNS	Same as xNF but the order of SMILES in reactants/reagents was randomly shuffled.
xNM	The same as xNS but also contained the same number of inverted (forward synthesis) reactions. The forward reactions started with "." to distinguish them from retro-synthetic ones.

Supplementary Table 2. Examples of data augmentations for two reactions. Canonical SMILES are shown in bold.

Data set	input (product) >> output (reactants)	Examples
x0	canonical >> canonical	**CC(c1ccc(Br)nc1)N(C)C >> CC(=O)c1ccc(Br)nc1.CNC**<br>**O=Cc1cncc(Br)c1 >> O=C(O)c1cncc(Br)c1**
x2	canonical >> canonical<br>random >> canonical	**CC(c1ccc(Br)nc1)N(C)C >> CC(=O)c1ccc(Br)nc1.CNC**<br>n1c(Br)ccc(c1)C(N(C)C)C >> **CC(=O)c1ccc(Br)nc1.CNC**<br><br>**O=Cc1cncc(Br)c1 >> O=C(O)c1cncc(Br)c1**<br>c1(cncc(Br)c1)C=O >> **O=C(O)c1cncc(Br)c1**
x2R	canonical >> fixed random<br>random >> fixed random	**CC(c1ccc(Br)nc1)N(C)C >>** c1cc(Br)ncc1C(=O)C.CNC<br>n1c(Br)ccc(c1)C(N(C)C)C >> c1cc(Br)ncc1C(=O)C.CNC<br><br>**O=Cc1cncc(Br)c1 >>** c1c(cncc1C(=O)O)Br<br>c1(cncc(Br)c1)C=O >> c1c(cncc1C(=O)O)Br
x2F	canonical >> canonical<br>random >> random	**CC(c1ccc(Br)nc1)N(C)C >> CC(=O)c1ccc(Br)nc1.CNC**<br>n1c(Br)ccc(c1)C(N(C)C)C >> CC(=O)c1ccc(nc1)Br.CNC<br><br>**O=Cc1cncc(Br)c1 >> O=C(O)c1cncc(Br)c1**<br>c1(cncc(Br)c1)C=O >> c1c(cncc1C(=O)O)Br
x3S	canonical >> canonical<br>random >> random+shuffled<br>random >> random+shuffled	**CC(c1ccc(Br)nc1)N(C)C >> CC(=O)c1ccc(Br)nc1.CNC**<br>n1c(Br)ccc(c1)C(N(C)C)C >> CNC.CC(=O)c1ccc(nc1)Br<br>CN(C(c1ccc(Br)nc1)C)C >> c1cc(Br)ncc1C(O)C.CNC<br><br>**O=Cc1cncc(Br)c1 >> O=C(O)c1cncc(Br)c1**<br>c1(cncc(Br)c1)C=O >> c1c(cncc1C(=O)O)Br<br>n1cc(cc(c1)C=O)Br >> OC(=O)c1cncc(c1)Br
x2M	canonical >> canonical<br>.canonical >> canonical<br>random >> random+shuffled<br>.random+shuffled >> random	**CC(c1ccc(Br)nc1)N(C)C >> CC(=O)c1ccc(Br)nc1.CNC**<br>**.CC(=O)c1ccc(Br)nc1.CNC >> CC(c1ccc(Br)nc1)N(C)C**<br>n1c(Br)ccc(c1)C(N(C)C)C >> CNC.CC(=O)c1ccc(nc1)Br<br>.CNC.CC(=O)c1ccc(nc1)Br >> n1c(Br)ccc(c1)C(N(C)C)C

		**O=Cc1cncc(Br)c1 >> O=C(O)c1cncc(Br)c1**<br>**.O=C(O)c1cncc(Br)c1 >> O=Cc1cncc(Br)c1**<br>c1(cncc(Br)c1)C=O >> c1c(cncc1C(=O)O)Br<br>.c1c(cncc1C(=O)O)Br >> c1(cncc(Br)c1)C=O

Analysis of predicted SMILES

The beam search was used to infer n=5 (or more) reactant sets from the model for each entry in the test file. The SMILES predicted within the same beam search were sorted in the decreasing order of their probabilities. Predictions containing erroneous SMILES representations, which could not be processed by RDKit, were discarded. The remaining predictions were converted to canonical SMILES. In cases where the predicted reaction contained several disconnected SMILES, they were sorted to have the same representation. If two or more identical predictions were found for the same input only the first prediction was kept: in this way we deduplicated reactions predicted for the same input data. For augmented test datasets, SMILES predicted for the same reaction were accumulated and those with the largest number of occurrences were selected as the top-ranked. If exactly the same number of predictions were found for two or more SMILES, the weights of the SMILES were set to be inversely proportional to their relative position in the respective beam search. Precisely, to rank predictions we used the following formula

$$rank(SMILES) = \sum_{n=0,..,\ augmentations} \sum_{i=1,..,\ beam} \delta(SMILES\{n,i\}, TARGET)/(1. + 0.001 * i)$$

where the first sum was over canonical (n=0) and augmented SMILES for the same input reaction. When the target canonicalized SMILES was equal to the predicted canonicalized SMILES at position *i* of the beam search for augmentation n, $\delta = 1$. Otherwise, if predicted and target SMILES did not coincide, $\delta = 0$. The term 0.001**i* was used to weight the predicted SMILES to be inversely proportional to its position in the beam search (see also Supplementary Tables 3 and 4).

Top-n performance accuracy

For the analysed input reaction we received a set of generated canonical SMILES (contributed both by beam search and augmentation procedure), which were ranked as explained above. If any of these top-n sequences coincided with the target canonical SMILES for the analysed reaction, the prediction was considered to be the correct one. The top-n accuracy was the ratio of the number of correct predictions to the total number of sequences in the test set.

Supplementary Table 3. Illustration of a procedure used to rank predicted reactions

Step	Input SMILES	Beam1,Beam2,Beam3
Initial prediction	SMILES_CAN<br>SMILES_AUG1<br>SMILES_AUG2	CC(C),C(C)CC,C(N)N<br>CNN,CCC,CC=<br>CC.CCC,CCC.CC,C#
Canonisation, sorting and error detection	SMILES_CAN<br>SMILES_AUG1<br>SMILES_AUG2	CCC,CCCC,CNN<br>CNN,CCC,error<br>CC.CCC,CC.CCC,error
Elimination of duplicates and errors	SMILES_CAN<br>SMILES_AUG1<br>SMILES_AUG2	CCC,CCCC,CNN<br>CNN,CCC<br>CC.CCC
Enumeration	SMILES_CAN<br>SMILES_AUG1<br>SMILES_AUG2	CCC(0),CCCC(1),CNN(2)<br>CNN(0),CCC(1)<br>CC.CCC(0)
Ranks, see eq. 1		CCC = [1] +[1/(1+1./1000)] + [0] = **1.999**<br>CNN = [1/(1+2./1000)] + [1] + [0] = 1.998<br>CC.CCC =[0] + [0] + [1] = 1<br>CCCC = [1/(1+1./1000)] + [0] + [0] = 0.999

The top-2 ranked predictions were CCC and CNN

Supplementary Table 4. Illustration of procedure used to rank predicted reactions when using multiple predictions within the same beam

Step	Input SMILES	Beam1,Beam2,Beam3
Initial prediction	SMILES_CAN<br>SMILES_AUG1<br>SMILES_AUG2	CC(C),C(C)CC,C(N)N<br>CNN,CCC,CC=<br>CC.CCC,CCC.CC,C#
Canonisation, sorting and error detection	SMILES_CAN<br>SMILES_AUG1<br>SMILES_AUG2	CCC,CCCC,CNN<br>CNN,CCC,error<br>CC.CCC,CC.CCC,error
Elimination of errors	SMILES_CAN<br>SMILES_AUG1<br>SMILES_AUG2	CCC,CCCC,CNN<br>CNN,CCC<br>CC.CCC, CC.CCC
Enumeration	SMILES_CAN<br>SMILES_AUG1<br>SMILES_AUG2	CCC(0),CCCC(1),CNN(2)<br>CNN(0),CCC(1)<br>CC.CCC(0), CC.CCC (1)
Ranks, see eq. 1		CCC = [1] +[1/(1+1./1000)] + [0] = **1.999**<br>CNN = [1/(1+2./1000)] + [1] + [0] = 1.998<br>CC.CCC =[0] + [0] + [1] + [1/(1+1./1000)] = **1.999**<br>CCCC = [1/(1+1./1000)] + [0] + [0] = 0.999

The top-2 ranked predictions were CCC and CC.CCC.

The SMILES strings with the largest weights and thus those that appeared most frequently amidst the first sequences within the beam predictions were selected as the top-ranked. The top-1 and top-5 SMILES were used to estimate the prediction performances of models.

Analysis of stereochemistry-free datasets

About 20% of the reactions in the training and test sets had molecules with stereochemistry. The stereochemistry was encoded in SMILES with “/”,”\”,”@” and “@@” characters. However, a number of practical projects have relaxed stereochemistry requirements. Therefore, we separately reported the performance of the models for datasets with and without stereo-chemical information.

Character and exact sequence performance during training

During the model training, we calculated character-based performance, which corresponded to the number of exactly predicted characters for the target SMILES, as well as exact sequence accuracy indicating the number of correctly predicted exact sequences. Both of these measures were approximations of the final accuracy, for which the predicted SMILES were first converted to canonical ones and only after were compared to the target values.

Table 5. Examples of distributions of predicted SMILES

Reaction	Frequency of SMILES	Ratio of the most frequent to all SMILES
CCOC(=O)C1CCCN(C(=O)COc2ccc(-c3ccc(C#N)cc3)cc2)C1>>CCOC(=O)C1CCCNC1.N#Cc1ccc(-c2ccc(OCC(=O)O)cc2)cc1	926* 51 7 6 2 1 1 1 1 1 1 1	926/999 = 0.93
CCCCC(=O)O>>CCCCC(=O)OC(=O)CCCC	203 112 107 98 57 19 16 13 12 12 12 12 11 11 11 11 11 11 11 11 11 11 8 8 8 8 6 6 6 6 6 6 6 6 5 5 5 5 5 5 5 5 5 4 4 4 4 4 3 3 3 3 3 3 3 3 3 3 3 3 3 3 3 3 3 3 2 2 2 2 2 2 1 1 1 1 1 1	203/999 = 0.2

Star indicates the correctly predicted reaction. For the first reaction the most frequent SMILES was predicted 926 times or 93% of all predictions. For this SMILES the Transformer was very confident in the outcome of retrosynthesis, which it correctly predicted. For the second reaction the Transformer generated 78 different SMILES and the top-1 SMILES appeared only in 20% of all predictions. For this reaction the Transformer failed to predict the correct SMILES at all.

Unless otherwise noticed, the results presented in the supplementary Figures were calculated using the USPTO-50k set.

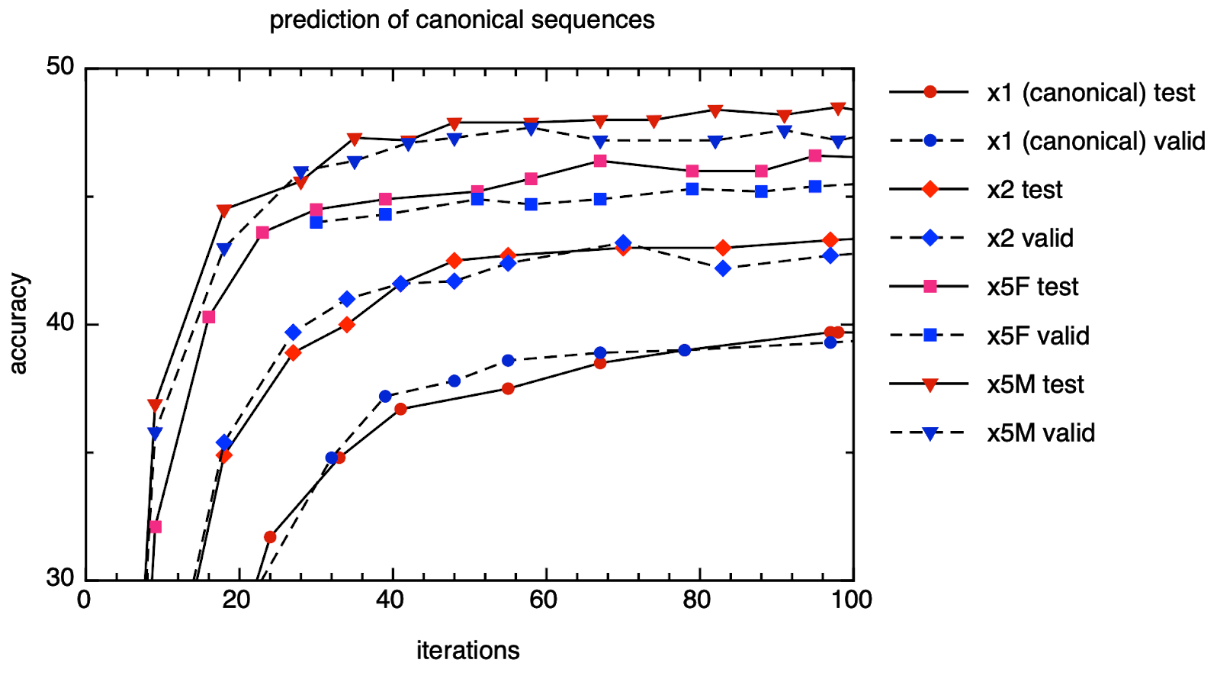

Supplementary Figure 1. Top-1 accuracies calculated for models developed with different augmentation scenarios and using 40k sequences as the training set. All models were applied to the x1 (canonical) test and validation set as they were defined in [10]. As we can see the performance of models is similar for both training and validation sets and it is monotonically increasing with the number of iterations. This observation was the main motivation to join training and validation sets as a single set, which was used for model development.

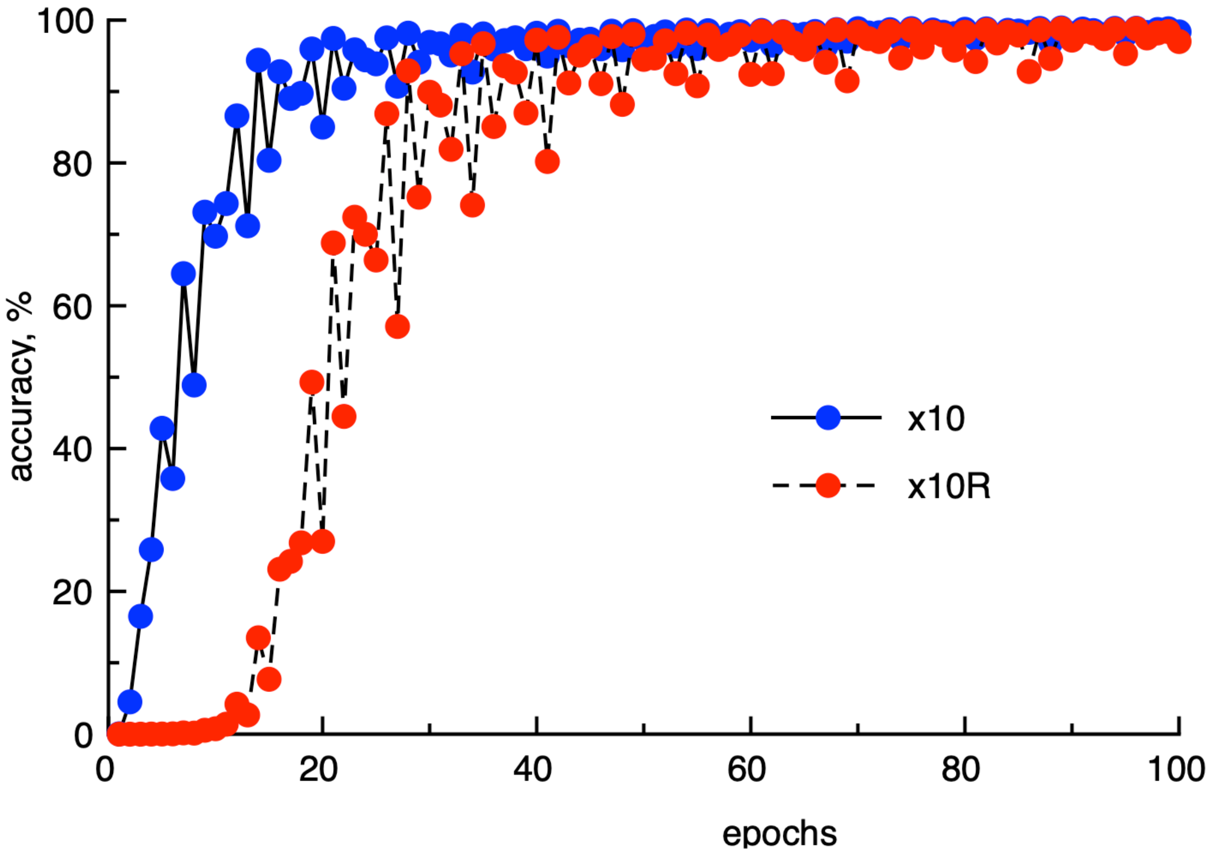

Supplementary Figure 2. Monitoring set accuracy (measured as a character accuracy) of Transformer for prediction of canonical (x10) and random (x10R) SMILES for USPTO-50k set (see Supplementary Tables 1 and 2 for explanation of used abbreviations).

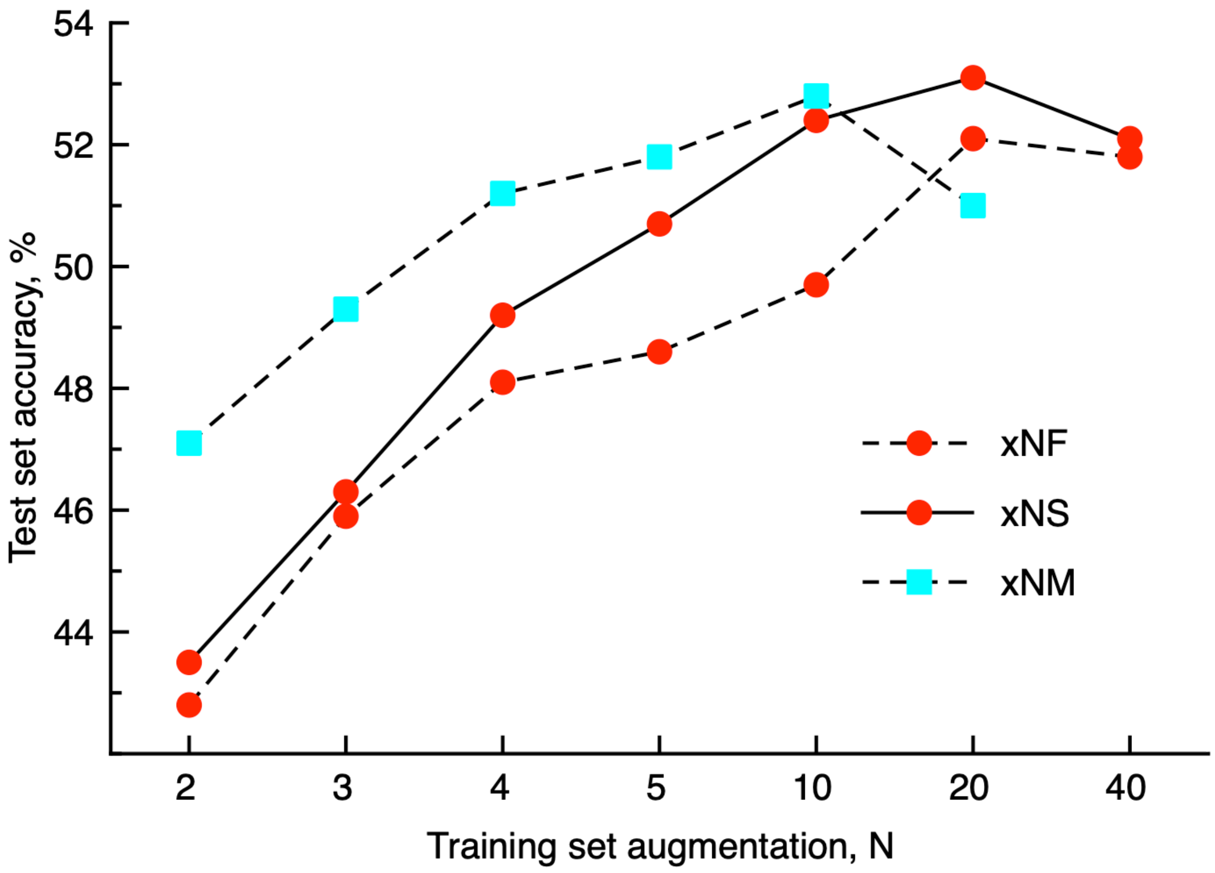

Supplementary Figure 3. Top-1 full-sequence retrosynthesis accuracies calculated for models developed with different augmentation scenarios for USPTO-50k training set. All models were applied to the x20 test set.

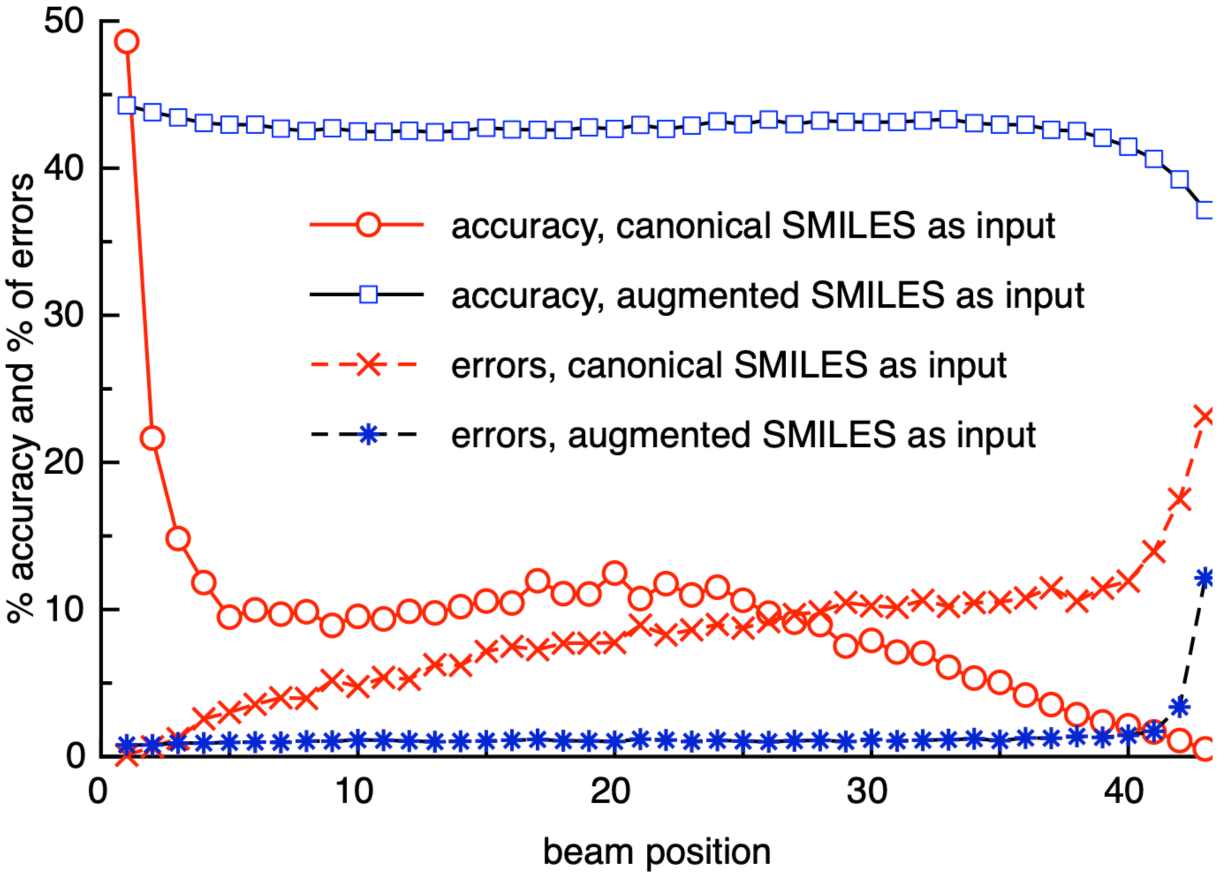

Supplementary Figure 4. Accuracy of prediction of SMILES generated at the respective position of the beam search using the largest beam size=44. The results were calculated for test set prediction using the model trained with 500 iterations on the USPTO-50k set. The use of canonical SMILES as input produced the highest accuracy (48.3%) for the first beam, which degraded for other positions of the beam while use of augmented SMILES provides about 44% correct predictions, which is slowly decreasing with the increase of the beam position. The number of erroneous SMILES is increasing with the beam position for both types of SMILES, but it was significantly higher for predictions when using canonical SMILES as input.

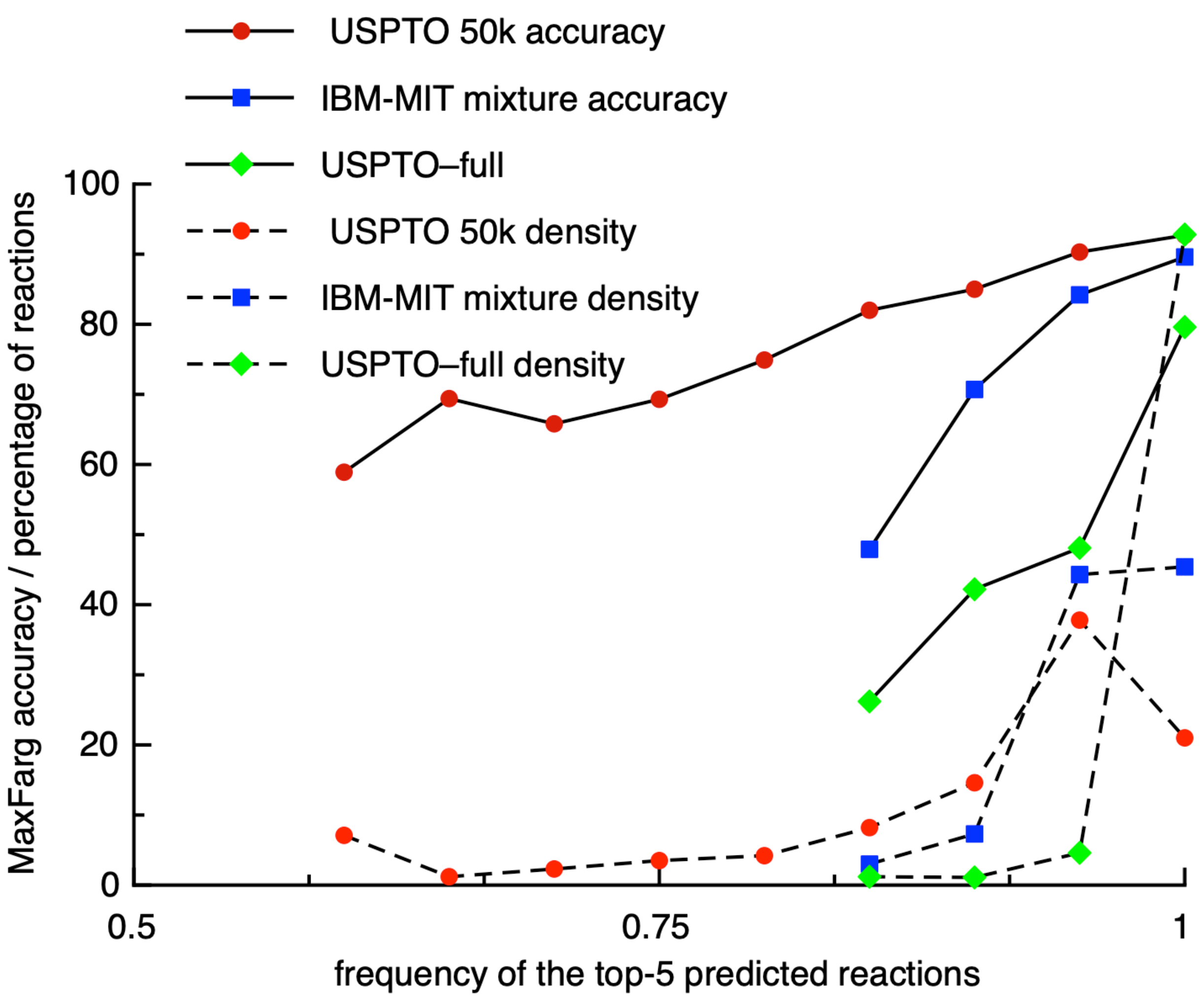

Supplementary Figure 5. Accuracy and density (fraction of predictions) of the Transformer for MaxFrag top-5 retrosynthesis accuracy as a function of the frequency of appearance of five most frequent SMILESes in the output of the Transformer (see also Figures in the article). Due to a small number of samples and high variability of data the average accuracy is shown for each first left datapoint for the same or smaller frequencies. For example for USPTO 50k set the accuracy of 58.9% for frequency 0.6 was calculated by averaging the MaxFrag accuracies for SMILES with frequencies ≤ 0.6. There were 7.1% of such predictions in the test dataset.

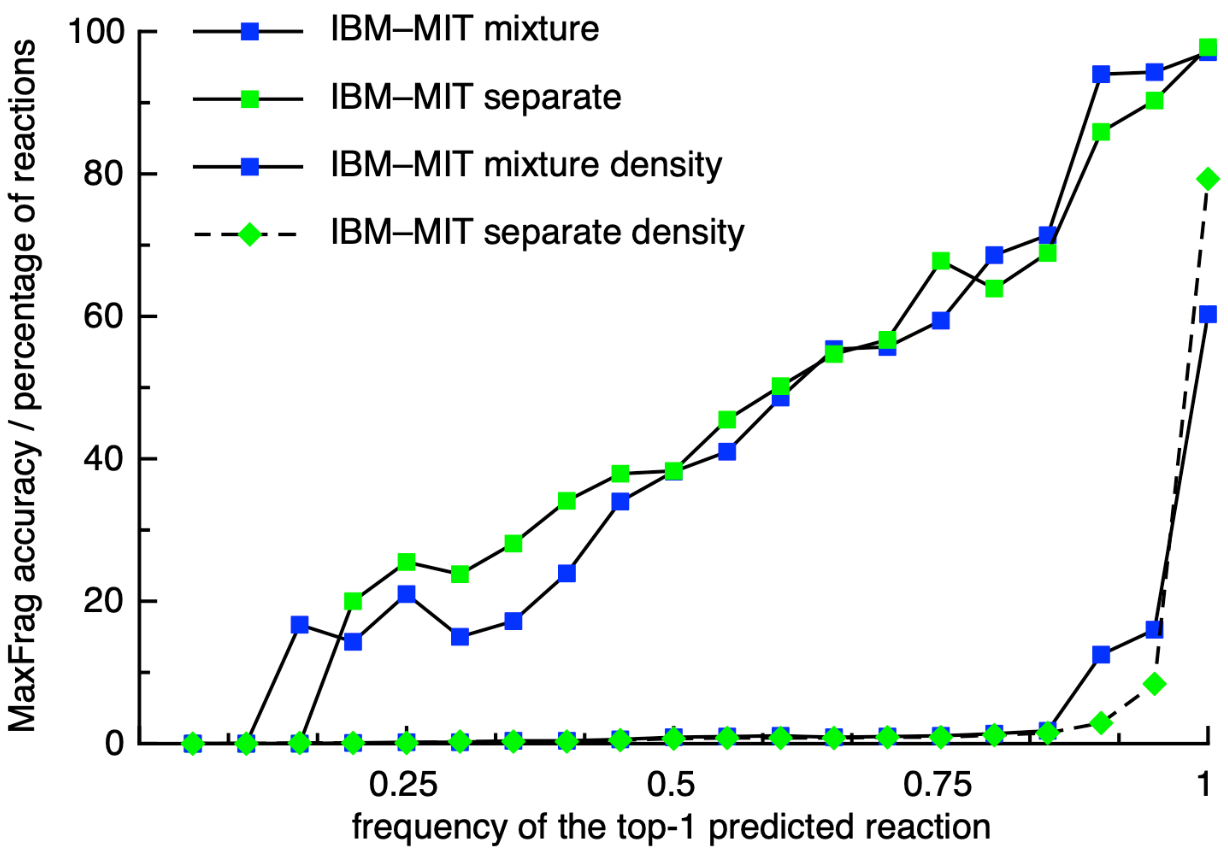

Supplementary Figure 6. Accuracy and density (fraction of predictions) of the Transformer for MaxFrag top-1 direct synthesis accuracy as a function of the frequency of appearance of the top-1 SMILES in the output of the Transformer for the respective test sets of the models (see also Figures in the article).

Calculation of the decrease of the relative errors

Let us assume that we can (theoretically) get 100% accuracy. Top-5 error of the previous model for the mixed set was 100-94.2 = 5.8%. The error of our model is 100 - 96.1 = 3.9%. The relative decrease of the error is (5.8-3.9)/5.8 = 32.7%.

Supplementary References

(1) Pavel Karpov, Guillaume Godin, Igor V. Tetko. A Transformer Model for Retrosynthesis. In *28th International Conference on Artificial Neural Networks, Munich, Germany, September 17–19, 2019 Proceedings, Part V, Workshop and Special sessions*; Igor V. Tetko, Fabian Theis, Pavel Karpov, Vera Kurkova, Ed.; Lecture Notes in Computer Science; Springer.

(2) Vaswani, A.; Shazeer, N.; Parmar, N.; Uszkoreit, J.; Jones, L.; Gomez, A. N.; Kaiser, L.; Polosukhin, I. Attention Is All You Need. *ArXiv* **2017**.

(3) Schwaller, P.; Gaudin, T.; Lányi, D.; Bekas, C.; Laino, T. "Found in Translation": Predicting Outcomes of Complex Organic Chemistry Reactions Using Neural Sequence-to-Sequence Models. *Chem. Sci.* **2018**, *9* (28), 6091–6098.

(4) Zheng, S.; Rao, J.; Zhang, Z.; Xu, J.; Yang, Y. Predicting Retrosynthetic Reactions Using Self-Corrected Transformer Neural Networks. *J. Chem. Inf. Model.* **2020**, *60* (1), 47–55.

(5) Karpov, P.; Godin, G.; Tetko, I. V. Transformer-CNN: Swiss Knife for QSAR Modeling and Interpretation. *J. Cheminform.* **2020**, *12* (1), 17.

(6) Britz, D.; Goldie, A.; Luong, M.-T.; Le, Q. Massive Exploration of Neural Machine Translation Architectures. *arXiv [cs.CL]*, 2017.

(7) Landrum, G. RDKit: Open-source cheminformatics http://www.rdkit.org.

(8) Tetko, I. V.; Karpov, P.; Bruno, E.; Kimber, T. B.; Godin, G. Augmentation Is What You Need!: 28th International Conference on Artificial Neural Networks, Munich, Germany, September 17–19, 2019, Proceedings. In *Artificial Neural Networks and Machine Learning – ICANN 2019: Workshop and Special Sessions*; Tetko, I. V., Kůrková, V., Karpov, P., Theis, F., Eds.; Lecture Notes in Computer Science; Springer International Publishing: Cham, 2019; Vol. 11731, pp 831–835.

(9) Kimber, T. B.; Engelke, S.; Tetko, I. V.; Bruno, E. Synergy Effect between Convolutional Neural Networks and the Multiplicity of Smiles for Improvement of Molecular Prediction. *arXiv preprint arXiv* **2018**.

(10) Liu, B.; Ramsundar, B.; Kawthekar, P.; Shi, J.; Gomes, J.; Luu Nguyen, Q.; Ho, S.; Sloane, J.; Wender, P.; Pande, V. Retrosynthetic Reaction Prediction Using Neural Sequence-to-Sequence Models. *ACS Central Science* **2017**, *3* (10), 1103–1113.
